# Histological and immunohistochemical outcomes after microdissection TESE in contrast with hormonal profile, testis volume and genetics in patients with azoospermia

**DOI:** 10.25122/jml-2022-0336

**Published:** 2023-01

**Authors:** Iurii Arian, Ion Dumbraveanu, Victoria Ghenciu, Daniela Machidon, Ion Ernu, Emil Ceban

**Affiliations:** 1 Department of Urology and Surgical Nephrology, Nicolae Testemiţanu State University of Medicine and Pharmacy, Chisinau, Moldova; 2 Laboratory of Andrology, Functional Urology and Sexual Medicine, Nicolae Testemiţanu State University of Medicine and Pharmacy, Chisinau, Moldova

**Keywords:** azoospermia, non-obstructive azoospermia, maturation arrest, Sertoli only cells syndrome, micro-TESE, conventional TESE, AZF microdeletions – azoospermia factor microdeletions, CFTR mutations – cystic fibrosis transmembrane conductance regulator, FSH – follicle-stimulating hormone, ICSI – intracytoplasmic sperm injection, IVF – *in vitro* fertilisation, LH – luteinizing hormone, Micro-TESE – microdissection testicular sperm extraction, NOA – non-obstructive azoospermia, OA – obstructive azoospermia, PBS – phosphate-buffered saline, PLAP – anti-placental alkaline phosphatase, SHBG – sex hormone binding globulin, SOCS – Sertoli only cells syndrome, SSR – successful sperm retrieval, TESE – testicular sperm extraction

## Abstract

A limited number of individuals with non-obstructive azoospermia (NOA) may recover spermatozoa through traditional testicular sperm extraction (TESE) techniques. There is an ongoing debate over the effectiveness of microdissection TESE compared to standard TESE methods. Microdissection TESE (micro-TESE) techniques enable the identification of spermatogenesis foci in non-obstructive forms of azoospermia. Only histological examination can provide an objective and definitive assessment of the testicular phenotype. This study aimed to evaluate the correlation between histopathological findings after microdissection TESE (micro-TESE) and the predictive role of various factors in determining the success of sperm retrieval. We evaluated 24 patients with azoospermia who underwent micro-TESE and considered the patient's hormonal profile, testis ultrasound, genetic evaluation, histology, and immunohistology (PLAP antibody) of collected testis biopsies. The preoperative blood FSH level, in conjunction with other parameters, may aid in the prediction of micro-TESE success. Sensitivity increases, and specificity decreases with higher FSH levels. Furthermore, testicular volume and FSH levels are typically normal in patients with maturation arrest. In conclusion, hormones, ultrasound evaluation of the testicles, testis volume, and available genetic tests have a predictive value in differentiating obstructive azoospermia (OA) from NOA with various sensitivity and specificity rates. Histological and immunohistochemical evaluation establishes the testicular phenotype accurately and guides patient management.

## INTRODUCTION

Non-obstructive azoospermia (NOA) is distinguished by a complete absence of spermatozoa in semen due to minimal or no spermatogenesis, as opposed to obstructive azoospermia (OA), which is defined by an obstruction in the ductal system. Genetic conditions such as sexual chromosomal abnormalities, Y chromosome translocations and microdeletions, cryptorchidism, testicular torsion, radiation, and toxins are potential causes [1, 2]. NOA accounts for about 10% of all male infertility [3]. Fine needle aspiration, which may be ultrasound-guided, traditional testicular sperm extraction (TESE), and microdissection TESE, are all alternatives for collecting viable spermatozoa in these patients. The first line of treatment for people with NOA is now TESE combined with intracytoplasmatic sperm injection. The recovery of spermatozoa in NOA appears to be more successful with testicular biopsy than with FNA [4, 5].

Conventional TESE was once considered the gold standard for sperm recovery in these individuals. However, the procedure involves exposing the testis by a tiny incision and taking one or more blind biopsies, with an average retrieval rate of about 50% in NOA patients [6]. Though rare, potential side effects could include hematoma, inflammatory changes, irreversible devascularization, and severe testicular tissue loss [7].

In 1999, MicroTESE was first made available [8]. In this method, the tunica albuginea is extensively opened, and the testicular tissue is examined under an operating microscope at a magnification of 20 to 25x, enabling the visualization of whitish, bigger, and more opaque tubuli. This method is based on the assumption that these tubuli have active spermatogenesis, and by identifying sub-tunical vessels, the risk of devascularization is reduced. Multiple studies have been conducted to compare the effectiveness of conventional TESE and micro-TESE.

Testicular volume and testicular germ cell content are reflected in serum FSH levels. Men may be diagnosed with NOA if their FSH level is less than 7.6 mIU/mL or their testicular long axis measures less than 4.6 cm [9]. If these men consent to advanced reproductive care, therapeutic testicular biopsy and sperm extraction, processing, and cryopreservation for IVF/ICSI are the recommended course of action [9].

A different, perhaps more effective strategy is required due to the high likelihood of failure, which includes the 30%–60% of men without sperm and the 50%–91% of IVF/ICSI couples who do not become pregnant [10–12]. Currently, there is a lack of consensus on the best approach for selecting patients and timing for surgery [13]. Although the effectiveness of micro-TESE in comparison to other sperm retrieval methods has been widely acknowledged, it is still unclear and controversial how to determine who may benefit from the technique prior to surgery [12].

## MATERIAL AND METHODS

The study included 24 patients, representing the first treatment experience using micro-surgical methods in patients with azoospermia in the Republic of Moldova. Patients underwent investigations according to the pre-established algorithm: sperm evaluation accordingly to the WHO 2010 guide recommendations, hormonal testing (LH, FSH, testosterone, SHBG, prolactin, and estradiol), ultrasound of the scrotum and relevant genetic examination (karyotype, AZF microdeletions, and CFTR mutations) before surgical treatment. Azoospermia was established when the absence of spermatozoa was confirmed in two semen samples. Thus, according to WHO guidelines, sediment analysis was performed after 600 g centrifugation for 15 minutes and screening at 400x magnification using an inverted microscope. The same team worked on all interventions and post-intervention tissue analyses. The tissue removed during microsurgery was carefully examined using a combination of mechanical and enzymatic maceration techniques. Samples were preserved by freezing and then subjected to both histological and immunohistochemical testing to obtain detailed information about the tissue.

### Surgical procedure

All patients underwent surgery according to the same algorithm and surgical technique. The surgical intervention was performed under spinal anesthesia. The tunica vaginalis is opened following a midline scrotal incision. The testis is opened widely in an equatorial plane in the middle, revealing the testis covered with the tunica albuginea. As a result, seminiferous tubules can be exposed widely in a natural manner that mimics intratesticular blood flow. The remaining steps of the operation are carried out under a 20–25x operating microscope. The tubules are removed for small samples. Sperm are more likely to be found in bigger and more opaque tubules. Depending on the size of the testicles and the condition of the tubules, up to 15 biopsies may be collected from each side. Once all visible parenchymal regions have been examined under a microscope or when additional dissection is deemed likely to endanger the testicular blood supply, the surgery is over. All the fragments were collected in 3 samples: sample 1 – intended for the preliminary control, carried out on the day of the intervention; sample 2 – intended for gradual cryo-freezing; and sample 3 – intended for the histological examination, which was taken and fixed in Bouin's solution.

### Preliminary evaluation of testicular tissue

After the initial samples were taken, the tissue was examined intraoperatively, and a preliminary assessment of the presence or lack of spermatozoa in the samples was made. Before centrifugation, the tissue was mechanically macerated and suspended in the washing solution (Sperm Air, Ginemed). The sediment was inspected under a phase contrast microscope at x200 magnification. The outcome was reported intraoperatively. If no sperm were discovered, the tissue was then exposed to the enzymatic lysis procedure. The collagenase solution and tissue suspension were combined in a 1:1 ratio, and the mixture was shaken every 10–15 minutes throughout an incubation period of 60 minutes at 37℃. After the incubation, the undigested tissue was pelleted, and the supernatant was separated using centrifugation at 50 g for 5 minutes. Enzymes were taken out by adding an equivalent amount of wash media. The supernatant was separated using two 1800 g/5-minute centrifugations to separate the sample. The sediment was checked with a micro drop on the day of the intervention, and the results were recorded.

### Testicular tissue freezing

The collected testicular tissue (2^nd^ sample) was frozen in order to preserve fertility and short-term storage until the histology results with the determination of the histological phenotype were available. Therefore, the testicular tissue was fixed using a special sperm-freezing media (Sperm Freezing Medium, ORIGIO, Denmark). Additionally, programmed freezing was carried out on the fixed tissue using freezing equipment (in our case Freeze Control CL 8800i, Cryologic, Australia). The recommended procedure consisted of cooling the tissue to -10℃ for the first 10 minutes, then cooling the content to -40℃ in the following 7 minutes before the samples were placed in liquid nitrogen storage. This was followed by a 5-minute plateau period.

### Histological and immunohistochemical examination

The tissue (3^rd^ sample) was fixed using Bouin's solution for 24 hours. The tissue in the fixative was dried after many rinses. Cleaning, embedding medium (paraffin) infiltration, microtome sectioning, transfer to slides, and drying the sections were performed after dehydration. It was then deparaffinized in xylene, rehydrated, stained with hematoxylin, thoroughly rinsed, stained with eosin, rinsed with ethanol, dehydrated, cleaned, and covered.

As previously mentioned, paraffin-embedded tissue blocks were sliced into pieces that were between three and five micrometers thick, and these sections were then put on silane-coated slides. Tissue section-containing slides were incubated for 20 minutes at 60℃. Following deparaffinization, the sections were heated for 3–5 minutes at 800 and 400 W in a microwave with 10 mmol/L of citrate buffer (pH 6.0) in order to retrieve antigens. The tissue slides were then rinsed with phosphate-buffered saline (PBS), and the endogenous peroxidase activity was suppressed by a 5-min treatment with H_2_O_2_. After being cleaned with PBS buffer, the slides were incubated with the primary antibody (placental alkaline phosphatase, PLAP) for 30 minutes at room temperature. The secondary biotinylated antibody was added for a 30-min incubation after washing in PBS. Following a PBS buffer wash, the slides were incubated with streptavidin-horseradish peroxidase for 30 minutes. After a second PBS wash, chromogen was applied to the tissue slices for five minutes. The slides were dehydrated with alcohol (96%), cleaned with xylene, rinsed in distilled water, stained with hematoxylin for one minute, washed with water, and mechanically covered. This protocol was simultaneously done with the positive control tissue slides.

### Testicular biopsy evaluation and scoring

The sections were evaluated according to the following morphological criteria [14]:

- Absence of seminiferous tubules (tubular sclerosis);
- Absence of germ cells (Sertoli cell only syndrome);
- Maturation arrest – spermatogenesis arrested at different stages (spermatogonia, spermatocytes, or spermatids);
- Hypospermatogenesis – all cell types up to spermatozoa are present, but there is a distinct decline in reproducing spermatogonia;
- Mixed atrophy can be global (present in all tubules) or focal, with a variable percentage of tubules displaying various stages of qualitatively and quantitatively limited spermatogenesis.

Spermatogenesis was assessed using Johnsen's score. The Johnsen criteria convert the cell profile found along the seminiferous tubules into a ten-point score system for assessing spermatogenesis. Johnsen scores range from 1 to 10, with 1 representing the complete absence of germ cells and 10 representing maximum spermatogenesis activity.

## RESULTS

The study included 24 patients with an average age of 34, ranging from 23 to 42 years old. All patients were diagnosed with azoospermia after at least 2 semen analyses. Surgery was performed with informed consent after fully explaining the risks and success rates to the patients. Before the intervention, 10 patients received chorionic gonadotropin to adjust their testosterone levels, which were initially below 8 mmol/L. The results of hormonal investigations, testicular volume, and genetic diagnosis can be found in Table 1.

**Table 1:** Hormonal, testis volume, and genetic findings in the studied group.

P	FSH (mIU/mL)	LH (mIU/mL)	Testosteron (nmol/L)	Right testis volume (ml)	Left testis volume (ml)	Cariotype	AZF micro-deletions	CFTR mutations
**Hypergonadotropic azoospermia**								
**1.**	15.3	8.9	7.4	8.3	10.7	46,XY	No deletions	Absent
**2.**	24.5	12.1	10.1	7.5	6.4	46,XY	No deletions	Absent
**3.**	11.7	15.9	5.4	10.9	7.2	46,XY	No deletions	Absent
**4.**	35.1	18.4	11.3	6.8	8.9	46,XY	No deletions	Absent
**5.**	30.5	9.4	15.6	10.4	7.3	46,XY	No deletions	Absent
**6.**	39.0	21.6	10.6	5.2	4.2	46,XY	No deletions	Absent
**7.**	12.2	11.9	13.5	14.6	10.4	46,XY	No deletions	Absent
**8.**	16.2	6.3	9.8	18.2	16.9	46,XY	sY254, sY255, sY1291, sY242	Absent
**9.**	48.3	23.9	7.7	7.3	8.7	46, XY	No deletions	Absent
**10.**	27.5	12.9	8.2	8.5	8.4	46,XY	No deletions	Absent
**11.**	19.7	6.9	14.9	14.9	7.2	46,XY	No deletions	Absent
**12.**	30.1	19.4	4.5	5.8	8.7	46,XY	No deletions	Absent
**13.**	34.5	23.4	5.8	9.4	7.3	46,XY	No deletions	Absent
**14.**	45.0	21.6	13.0	6.2	4.6	46,XY	No deletions	Absent
**15.**	15.2	10.9	16.9	12.6	9.4	46,XY	No deletions	Absent
**16.**	34.2	17.3	8.7	3.4	3.0	47,XXY	No deletions	Absent
**Normogonadotropic azoospermia**								
**17.**	3.9	4.4	14.7	15.5	14.3	46,XY	No deletions	Absent
**18.**	4.3	3.3	19.1	16.7	18.3	46,XY	No deletions	Absent
**19.**	6.3	4.5	17.2	20.4	15.4	46,XY	No deletions	Absent
**20.**	7.1	5.2	16.5	14.5	13.2	46,XY	No deletions	Absent
**21.**	3.4	6.4	18.3	14.5	12.3	46,XY	No deletions	Absent
**22.**	5.2	2.3	16.0	18.7	19.3	46,XY	No deletions	Absent
**23.**	2.3	4.5	15.3	20.4	none	46,XY	No deletions	Absent
**24.**	7.8	8.2	16.3	16.5	15.2	46,XY	No deletions	Absent

Hypergonadotropic hypogonadism (FSH >8 mIU/mL or/and LH >7.6 mIU/mL) was confirmed in 16 patients (66.7%), while 8 patients (34.3%) were diagnosed with normogonadotropic hypogonadism. In patients with non-obstructive azoospermia, the average value of FSH was 27.4 mIU/mL, with values ranging from 11.7 and 48.3 mIU/mL. Only 6 patients (37.5%) in the hypergonadotropic hypogonadism group had FSH values <20 mIU/mL, which is considered the upper limit of FSH with a higher success rate for sperm retrieval. In the NOA group, only one patient had sperm cells, with an FSH value of 19.7 mIU/mL. The average value of LH in the NOA group was 15.05 mIU/mL, with values ranging from 6.3 and 23.4 mIU/mL. The average value of FSH and LH in patients with a presumptive diagnosis of obstructive azoospermia was 5.0 mIU/mL and 4.85 mIU/mL, respectively. The average testosterone value in patients with NOA was 10.2 nmol/L compared to 16.7 nmol/L in those with OA.

The average testicular volume in patients with NOA was 9.4 ml and 8 ml on the left and right, respectively. In patients with OA, the testicular volume was significantly increased, with an average value of 17.5 ml on the right and 15.4 on the left. In one patient, the only surgical testicle on the right was operated on; the left was removed in childhood for unknown reasons.

Genetic investigations revealed that 2 patients had genetic issues: one had Klinefelter syndrome (47, XXY) and one had an incomplete AZF microdeletion of the Y chromosome (type b and c). Additionally, all patients were tested for cystic fibrosis gene mutations with the absence of mutations in the study group. At the same time, a single patient was identified intra-operatively with bilateral vas deferens atresia, with confirmed obstructive azoospermia and the absence of cystic fibrosis gene mutations.

**Figure 1:**
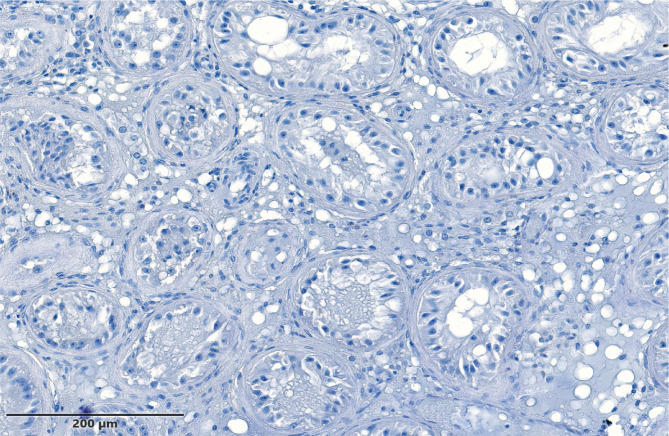
Sertoli only Cells Syndrome – most of the tubules can be found with a total absence of the germ cells and their precursors.

**Figure 2:**
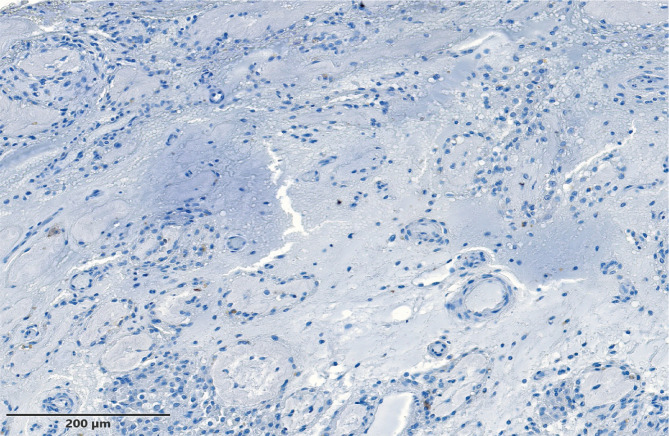
Testicular fibrosis – complete testicular fibrosis found in a patient with Klinefelter Syndrome.

**Figure 3:**
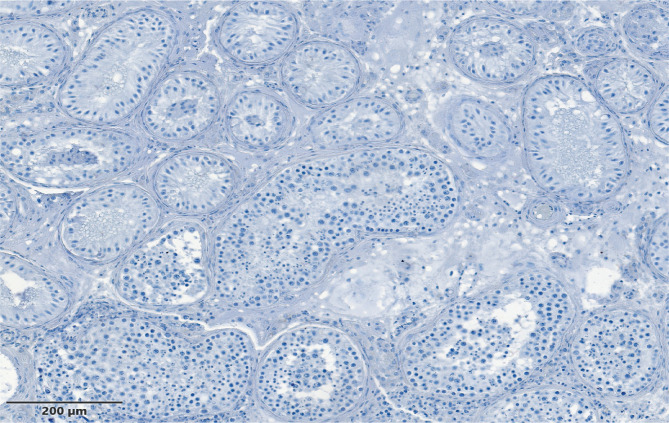
Mixt atrophy – completely normal tubules, with Johnsen score 9–10, near the Sertoli-only Cells Syndrome tubules and testicular fibrosis.

The results of the histological and immunohistochemical investigations are presented in Table 2. A total of 47 biopsies were obtained from 24 patients, with one surgical testicle biopsy taken from one patient. In the group of patients with hypergonadotropic hypogonadism, 8 patients (50%) were identified with Sertoli cell syndrome only (Figure 1), 4 patients (25%) with fibrosis and SOCS, and only one patient was identified with total fibrosis (Figure 2) of the testicular parenchyma, this patient was diagnosed with Klinefelter syndrome. Mixed atrophy (Figure 3) was identified in 2 patients with a pre-intervention diagnosis of NOA, and these were the only patients in whom spermatozoa were identified after the intervention. Maturation arrest was identified in only one patient from the group of patients with NOA, who was also diagnosed with Y chromosome microdeletion type b and c, with normal testicular volume, normal LH, and FSH value of 16.2 mIU/mL.

**Table 2:** Histological and immunohistological findings, Johnsen score, and sperm retrieval results.

P	Histology	Imunohistochemistry PLAP	Johnsen's score	Sperm retrival
**Hipergonadotropic azoospermia**				
**1.**	Fibrosis, SCOS	Negative	1-2	None
**2.**	SCOS	Negative	2	None
**3.**	Mixed atrophy: SCOS, fibrosis, normal morphology	Negative	1-9	Succesful
**4.**	Fibrosis, SCOS	Negative	1-2	None
**5.**	SCOS	Negative	2	None
**6.**	SCOS	Negative	2	None
**7.**	Fibrosis, SCOS	Negative	1-2	None
**8.**	Maturation arest, spermatide stage	Negative	2-7	None
**9.**	SCOC	Negative	2	None
**10.**	SCOC	Negative	2	None
**11.**	**Mixed atrophy: SCOS, fibrosis, normal morphology**	**Negative**	**1-9**	**Succesful**
**12.**	SCOC	Negative	2	None
**13.**	SCOS	Negative	2	None
**14.**	Fibrosis	**Negative**	1	None
**15.**	Fibrosis, SCOS	Negative	1-2	None
**16.**	**SCOS**	**Negative**	**2**	**None**
**Normogonadotropic azoospermia**				
**17.**	Normal morphology	Not done	9-10	Succesful
**18.**	Normal morphology	Not done	9-10	Succesful
**19.**	Normal morphology	Not done	9-10	Succesful
**20.**	Mixed atrophy: SCOS, maturation arest, normal morphology	Not done	1-9	None
**21.**	Maturation arest, spermatide stage	Not done	6-7	None
**22.**	Normal morphology	Not done	9-10	Succesful
**23.**	Normal morphology	Not done	9-10	Succesful
**24.**	Mixed atrophy: SCOS, maturation arest, normal morphology	Not done	1-9	Succesful

**Figure 4:**
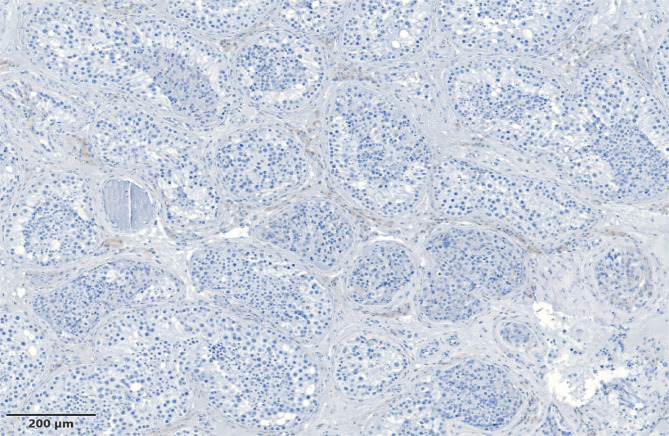
Obstructive azoospermia - multiple tubules with normal spermatogenesis, Johnsen score 9–10.

From the group of patients with OA, only 5 were confirmed with completely normal morphology of the tubules (Figure 4), and in 2 patients, the histological phenotype of mixed atrophy was established. A patient with absolutely normal testicular volume and hormonal values within the normal limits, presumed to be a patient with OA, was identified with a histological phenotype of maturation arrest with spermatids stage.

Patients with hypergonadotropic hypogonadism and NOA were additionally tested using immunohistochemistry techniques for PLAP antigen. All tested patients had negative results, the control sample being positive in all cases.

Histological samples were evaluated using Johnsen's score criteria to determine the presence or absence of germline cells. Thus, the maximum Johnsen score of 9–10 points was assessed in only 5 patients out of 8 presumed to have OA. Only one patient had a score of 6–7, specific to maturation arrest, and 2 other patients had a score of 1–9, indicating mixed atrophy. From the group of patients with NOA, the Johnsen score 1–2 was assigned to 13 patients, one patient was assigned a score of 1, indicating complete fibrosis, and the other 2 patients were assigned a score of 1–9, specific to mixed atrophy.

## DISCUSSIONS

While traditional TESE is a blind operation, microTESE has the potential advantage of allowing direct visualization of testicular tissue during the procedure. Unfortunately, there have not been any properly randomized comparative studies to date that compare microTESE to standard TESE in patients with non-obstructive azoospermia (NOA) due to the complexity of the study design. However, the published retrospective and prospective pseudo-randomized studies covering both methods generally demonstrate increased SRR after microTESE. It is improbable that the choice of patients has anything to do with a better outcome. Nevertheless, it is difficult to reach firm conclusions due to the paucity of studies contrasting the two approaches. The microTESE technique appears to be particularly beneficial for patients with Sertoli-only cells syndrome, as it allows for the identification of isolated areas of spermatogenesis, which may be the reason for the higher success rate in these patients [6]. It is generally known that some men with NOA still have patchy spermatogenesis, whereas other men with NOA lack germ cells and/or fail to mature spermatozoa in all tubuli.

Contrary to popular belief, patchy spermatogenesis may still be present in people with a histological diagnosis of SCOS. This discovery explains why the results of traditional TESE cannot be predicted by inadequate histology categorization. Furthermore, two studies have shown that microTESE in patients with Sertoli-only cells syndrome (SCOS) results in a higher sperm retrieval rate, suggesting that the use of microscopic magnification during the procedure allows for the identification of isolated areas of spermatogenesis in these patients.

MicroTESE appears less advantageous in maturation arrest, where all tubuli are microscopically homogeneous despite active spermatogenesis [15]. It is debatable whether serum FSH levels can accurately predict successful sperm retrieval, as studies have shown that higher FSH levels tend to result in lower success rates for both MicroTESE and other procedures. A major retrospective study showed that NOA males with elevated FSH have an equal chance of sperm retrieval as men with reduced FSH [16, 17]. Because spermatogenesis is irregular, the testicular volume has a poor predictive value. After the microTESE surgery, fewer sonographic problems were seen, including fibrosis and hematoma. However, given that no discernible difference in the rate of clinical complications is mentioned in any of the articles included, these findings appear to have little clinical significance. The serum testosterone levels revert to their initial levels following both treatments.

Several studies have been conducted to examine the use of preoperative factors in predicting sperm retrieval outcomes using conventional methods. Conventional TESE has been found to result in marked differences in FSH levels between patients where sperm retrieval is successful and those where it is not [18, 19]. Additionally, azoospermic men with high FSH have been demonstrated to have decreased sperm retrieval rates [20]. Several researchers have proposed that the administration of recombinant FSH prior to a sperm retrieval operation may improve the success rate of the procedure [21]. A study was conducted on 108 males with non-obstructive azoospermia (NOA) and normal FSH levels, where the study group received pure FSH therapy three times a week for three months prior to micro-TESE, while the control group received no therapy. The results of this study suggest that the preoperative FSH level, in combination with other parameters, may be useful in predicting the success of micro-TESE [22]. The sensitivity of FSH levels ranged from 9% to 71%, while specificity ranged from 40% to 90%.

Testicular volume cut-off values as low as 5 mL ranged from 7.6% to 50% in sensitivity and 6.7% to 71% in specificity. The sensitivity of FSH in predicting effective testicular sperm extraction (TESE) is poor within the range of normal FSH values. Sensitivity increases and specificity decreases with higher FSH levels [22].

Furthermore, testicular volume and FSH levels are typically normal in patients with maturation arrest [22, 23]. In other studies, patients with a high FSH level and a tiny testicle (26 mIU/mL and 5 mL, respectively) had spermatozoa or mature spermatids extracted from them or described in testicular biopsies [23–25]. More recently, sperm retrieval rates for TESE attempts in 42 men with Klinefelter syndrome and mean FSH levels of 33.2 IU/L were 72% [26].

However, most research indicates that FSH levels have minimal or no predictive value for the success of TESE and other sperm retrieval procedures [27–29], except for these outlier reports. In terms of micro-TESE, it was previously demonstrated that males with higher FSH levels had comparable or superior sperm retrieval to those with lower FSH levels [16, 30]. It is important to note that even among males with normal FSH levels, there may be instances where a consistent histological pattern of maturation arrest is present, resulting in extremely low rates of sperm retrieval [31].

Previous research has established a correlation between elevated FSH levels and poor histological characteristics, as well as lower rates of sperm retrieval in the testicular biopsy. [16, 32] These apparent inconsistencies can be explained by the fact that FSH serves as an indicator of overall testicular function. Since only a few seminiferous tubules in the testis may contain sperm, a high FSH level may not accurately reflect the amount of germ cells in the testis [33]. The utilization of micro-TESE has demonstrated the ability to achieve successful sperm retrieval in individuals with high levels of follicle-stimulating hormone (FSH). This surgical technique allows for the identification of regions of advanced spermatogenesis within the testicle, thereby increasing the likelihood of obtaining viable sperm. High or abnormal FSH levels should not be considered as a contraindication for the application of micro-TESE, particularly when performed by a skilled and experienced practitioner. In perspective, new factors for male infertility causes are expected to be considered for better prediction of SSR rate [34].

## CONCLUSIONS

The probability of successfully recovering sperm during surgery is complex and cannot be accurately predicted by any single factor or variable. Despite this, recent research suggests that a combination of preoperative factors can be used to inform patient counseling and guide clinical decision-making. However, more research is needed to develop a comprehensive strategy for predicting the outcomes of sperm retrieval in men with non-obstructive azoospermia (NOA) using genetic, molecular, and imaging techniques.

## References

[1] Schlegel PN, Sigman M, Collura B, De Jonge CJ (2021). Diagnosis and Treatment of Infertility in Men: AUA/ASRM Guideline PART II. J Urol.

[2] Esteves SC (2015). Clinical management of infertile men with nonobstructive azoospermia. Asian J Androl.

[3] Minhas S, Bettocchi C, Boeri L, Capogrosso P (2021). European Association of Urology Guidelines on Male Sexual and Reproductive Health: 2021 Update on Male Infertility. Eur Urol.

[4] El-Haggar S, Mostafa T, Abdel Nasser T, Hany R, Abdel Hadi A (2008). Fine needle aspiration *vs*. mTESE in non-obstructive azoospermia. Int J Androl.

[5] Bernie AM, Mata DA, Ramasamy R, Schlegel PN (2015). Comparison of microdissection testicular sperm extraction, conventional testicular sperm extraction, and testicular sperm aspiration for nonobstructive azoospermia: a systematic review and meta-analysis. Fertil Steril.

[6] Donoso P, Tournaye H, Devroey P (2007). Which is the best sperm retrieval technique for non-obstructive azoospermia? A systematic review. Hum Reprod Update.

[7] Schlegel PN, Su LM (1997). Physiological consequences of testicular sperm extraction. Hum Reprod.

[8] Majzoub A, Arafa M, Khalafalla K, AlSaid S (2021). Predictive model to estimate the chances of successful sperm retrieval by testicular sperm aspiration in patients with nonobstructive azoospermia. Fertil Steril.

[9] Deruyver Y, Vanderschueren D, Van der Aa F (2014). Outcome of microdissection TESE compared with conventional TESE in non-obstructive azoospermia: a systematic review. Andrology.

[10] Esteves SC, Ramasamy R, Colpi GM, Carvalho JF, Schlegel PN (2020). Sperm retrieval rates by micro-TESE *versus* conventional TESE in men with non-obstructive azoospermia-the assumption of independence in effect sizes might lead to misleading conclusions. Hum Reprod Update.

[11] Nicopoullos JD, Gilling-Smith C, Almeida PA, Norman-Taylor J (2004). Use of surgical sperm retrieval in azoospermic men: a meta-analysis. Fertil Steril.

[12] Achermann APP, Pereira TA, Esteves SC (2021). Microdissection testicular sperm extraction (micro-TESE) in men with infertility due to nonobstructive azoospermia: summary of current literature. Int Urol Nephrol.

[13] Corona G, Minhas S, Giwercman A, Bettocchi C (2019). Sperm recovery and ICSI outcomes in men with non-obstructive azoospermia: a systematic review and meta-analysis. Hum Reprod Update.

[14] Tüttelmann F, Ruckert C, Röpke A (2018). Disorders of spermatogenesis: Perspectives for novel genetic diagnostics after 20 years of unchanged routine. Med Genet.

[15] Bernie AM, Shah K, Halpern JA, Scovell J (2015). Outcomes of microdissection testicular sperm extraction in men with nonobstructive azoospermia due to maturation arrest. Fertil Steril.

[16] Ramasamy R, Lin K, Gosden LV, Rosenwaks Z (2009). High serum FSH levels in men with nonobstructive azoospermia does not affect success of microdissection testicular sperm extraction. Fertil Steril.

[17] Ramasamy R, Schlegel PN (2007). Microdissection testicular sperm extraction: effect of prior biopsy on success of sperm retrieval. J Urol.

[18] Mitchell V, Robin G, Boitrelle F, Massart P (2011). Correlation between testicular sperm extraction outcomes and clinical, endocrine and testicular histology parameters in 120 azoospermic men with normal serum FSH levels. Int J Androl.

[19] Boitrelle F, Robin G, Marcelli F, Albert M (2011). A predictive score for testicular sperm extraction quality and surgical ICSI outcome in non-obstructive azoospermia: a retrospective study. Hum Reprod.

[20] Zitzmann M, Nordhoff V, von Schönfeld V, Nordsiek-Mengede A (2006). Elevated follicle-stimulating hormone levels and the chances for azoospermic men to become fathers after retrieval of elongated spermatids from cryopreserved testicular tissue. Fertil Steril.

[21] Laursen RJ, Elbaek HO, Povlsen BB, Lykkegaard J (2019). Hormonal stimulation of spermatogenesis: a new way to treat the infertile male with non-obstructive azoospermia?. Int Urol Nephrol.

[22] Tsujimura A, Matsumiya K, Miyagawa Y, Takao T, Fujita K, Koga M, Takeyama M, Fujioka H, Okuyama A (2004). Prediction of successful outcome of microdissection testicular sperm extraction in men with idiopathic nonobstructive azoospermia. J Urol.

[23] Martin-du-Pan RC, Bischof P (1995). Increased follicle stimulating hormone in infertile men. Is increased plasma FSH always due to damaged germinal epithelium?. Hum Reprod.

[24] Devroey P, Liu J, Nagy Z, Goossens A (1995). Pregnancies after testicular sperm extraction and intracytoplasmic sperm injection in non-obstructive azoospermia. Hum Reprod.

[25] Carpi A, Fabris GF, Chiechi A, Nardini V (2002). Spermatogenesis in azoospermic, formerly cryptorchid men. Use of needle aspiration techniques. Acta Cytol.

[26] Schiff JD, Palermo GD, Veeck LL, Goldstein M (2005). Success of testicular sperm injection and intracytoplasmic sperm injection in men with Klinefelter syndrome. J Clin Endocrinol Metab.

[27] Huang X, Bai Q, Yan LY, Zhang QF (2012). Combination of serum inhibin B and follicle-stimulating hormone levels can not improve the diagnostic accuracy on testicular sperm extraction outcomes in Chinese non-obstructive azoospermic men. Chin Med J (Engl).

[28] Althakafi SA, Mustafa OM, Seyam RM, Al-Hathal N, Kattan S (2017). Serum testosterone levels and other determinants of sperm retrieval in microdissection testicular sperm extraction. Transl Androl Urol.

[29] Goulis DG, Polychronou P, Mikos T, Grimbizis G (2008). Serum inhibin-B and follicle stimulating hormone as predictors of the presence of sperm in testicular fine needle aspirate in men with azoospermia. Hormones (Athens).

[30] Jezek D, Knuth UA, Schulze W (1998). Successful testicular sperm extraction (TESE) in spite of high serum follicle stimulating hormone and azoospermia: correlation between testicular morphology, TESE results, semen analysis and serum hormone values in 103 infertile men. Hum Reprod.

[31] Hung AJ, King P, Schlegel PN (2007). Uniform testicular maturation arrest: a unique subset of men with nonobstructive azoospermia. J Urol.

[32] Chen CS, Chu SH, Lai YM, Wang ML, Chan PR (1996). Reconsideration of testicular biopsy and follicle-stimulating hormone measurement in the era of intracytoplasmic sperm injection for non-obstructive azoospermia?. Hum Reprod.

[33] Abdel Raheem A, Garaffa G, Rushwan N, De Luca F (2013). Testicular histopathology as a predictor of a positive sperm retrieval in men with non-obstructive azoospermia. BJU Int.

[34] Barbu MG, Thompson DC, Suciu N, Voinea SC (2021). The Roles of MicroRNAs in Male Infertility. Int J Mol Sci.

